# Phenol-soluble modulins α are major virulence factors of *Staphylococcus aureus* secretome promoting inflammatory response in human epidermis

**DOI:** 10.1080/21505594.2021.1975909

**Published:** 2021-09-13

**Authors:** Alexia Damour, Brandon Robin, Luc Deroche, Lauranne Broutin, Nicolas Bellin, Julien Verdon, Gérard Lina, Franck Marie Leclère, Magali Garcia, Julie Cremniter, Nicolas Lévêque, Charles Bodet

**Affiliations:** a Laboratoire Inflammation Tissus Epithéliaux et Cytokines EA 4331, Université De Poitiers, Poitiers, France; b Laboratoire De Bactériologie, CHU de Poitiers, Poitiers, France; c Laboratoire Ecologie et Biologie des Interactions, UMR CNRS 7267, Université De Poitiers, Poitiers, France; d CIRI Centre International de Recherche en Infectiologie, Inserm U1111, Université Lyon 1, Ecole Normale Supérieure de Lyon, Lyon, France; e Centre National de Référence des Staphylocoques, Institut des Agent Infectieux, Hôpital de La Croix Rousse, Hospices Civils de Lyon, Lyon, France; f Département de Chirurgie Plastique, Reconstructive et Esthétique, CHU de Poitiers, Poitiers, France; g Laboratoire de Virologie et Mycobactériologie, CHU de Poitiers, Poitiers, France

**Keywords:** *Staphylococcus aureus*, atopic dermatitis, phenol soluble modulins, inflammatory response, keratinocyte, epidermis, toxins, PSM α3

## Abstract

*Staphylococcus aureus* is a skin commensal microorganism commonly colonizing healthy humans. Nevertheless, *S. aureus* can also be responsible for cutaneous infections and contribute to flare-up of inflammatory skin diseases such as atopic dermatitis (AD), which is characterized by dysbiosis of the skin microbiota with *S. aureus* as the predominant species. However, the role of major virulence factors of this pathogen such as phenol-soluble modulin (PSM) toxins in epidermal inflammation remains poorly understood. Stimulation of primary human keratinocytes with sublytic concentrations of synthetic and purified PSM α3 resulted in upregulation of a large panel of pro-inflammatory chemokine and cytokine gene expression, including CXCL1, CXCL2, CXCL3, CXCL5, CXCL8, CCL20, IL-1α, IL-1β, IL-6, IL-36γ and TNF-α, while inducing the release of CXCL8, CCL20, TNF-α and IL-6. In addition, using *S. aureus* culture supernatant from mutants deleted from genes encoding either α-type PSMs or all PSM production, PSMs were shown to be the main factors of *S. aureus* secretome responsible for pro-inflammatory mediator induction in human keratinocytes. On the other hand, α-type PSM-containing supernatant triggered an intense induction of pro-inflammatory mediator expression and secretion during both topical and basal layer stimulation of an *ex vivo* model of human skin explants, a physiologically relevant model of pluristratified epidermis. Taken together, the results of this study show that PSMs and more specifically α-type PSMs are major virulence factors of *S. aureus* inducing a potent inflammatory response during infection of the human epidermis and could thereby contribute to AD flare-up through exacerbation of skin inflammation.

## Introduction

*Staphylococcus aureus* naturally colonizes the nares, pharynx, gastrointestinal tract, vagina, and skin of about 12% to 30% of healthy humans [1–3]. This commensal bacterium can become harmful being responsible for skin and soft tissue infections as well as invasive infections such as endocarditis, osteomyelitis or sepsis [1,2]. In addition, *S. aureus* is suspected of contributing to exacerbation of atopic dermatitis (AD) [4,5].

AD is a major inflammatory skin disease affecting about 20% of children and 1% to 5% of adults worldwide [6,7]. Physiopathology of AD is complex, involving many factors such as skin barrier dysfunction, and a predominant Th2 immune response associated with increased expression of interleukin (IL)-4 and IL-13 and dysbiosis of skin microbiota [6–8]. Indeed, in AD patients, microbiota diversity is strongly reduced for the benefit of *S. aureus*, which colonizes almost 90% of AD patients and represents about 65% of bacteria in AD antecubital and popliteal crease lesions during flares [3,9–11]. As compared to healthy patients, *S. aureus* colonization of AD patients is easier due to skin barrier disruption and enhanced expression of various protein targets for bacterial adhesion such as fibronectin and fibrinogen in the *stratum corneum* [9,12,13]. At that site, *S. aureus* can produce a huge amount of secreted toxins such as superantigens, especially enterotoxins, α-toxin, and phenol-soluble modulins (PSMs).

Among the secreted toxins of *S. aureus*, PSMs are considered as key virulence factors. PSMs form an amphipathic α-helix and are classified according to their length into α-type (20 to 24 amino acids) and β-type (43 to 45 amino acids) [14,15]. *S. aureus* produces four α-type PSMs (α1 to α4), two PSMs β (β1 and β2) and the δ-toxin (also known as PSMγ), which has a structure similar to α-type PSMs. These toxins are produced in high abundance, representing up to about 60% of the secreted protein mass [14–17]. Like many other virulence factors, PSM expression is strictly regulated by the quorum sensing system accessory gene regulator (Agr). Agr involves RNAIII, which controls transcription of virulence factors and of four *agr*A-D genes. Nevertheless, PSM expression regulation is independent of RNAIII. In fact, PSM expression is dependent on AgrA, which directly binds on the PSM promoters to induce their expression [17,18]. PSMs are produced with an N-terminal N-formylmethionine and their secretion is dependent on a noncanonical export system called pmt (phenol-soluble modulin transporter) and the multidrug resistance transporter AbcA [15,16,19]. However, in bacterial cytoplasm, the N-deformylase may cleave the N-terminal N-formyl group also resulting in secretion of a non-formylated form of PSM, and this deformylation step can modify the PSM biological activity [20]. Indeed, on neutrophils, N-formylated PSMs exert higher chemotactic activity than non-formylated PSMs [21].

PSMs, especially α-type PSMs, can play a key role in *S. aureus* pathogenesis. It was recently reported that these toxins contribute to the pathophysiology of central nervous system infections by triggering bacterial cell aggregation and the pathophysiology of bone and joint infections through induction of cytotoxicity and DNA damage in osteoblasts [22–24]. At high concentrations, PSMs are able to induce lysis of neutrophils, erythrocytes, mastocytes and keratinocytes by forming pores in the cell membrane [17,25–27]. Whatever the cell type, PSM α3, appears to be the most potent [17,26]. PSM α3 is able to form cross-α fibrils, which co-aggregate with the cell membrane, resulting in membrane rupture and T cell death [28]. And given their surfactant properties, PSMs are involved in surface colonization of skin or biomedical material and contribute to the structure and the dissemination of biofilms [14,15,29–31]. At lower concentrations, PSMs promote chemotaxis, activation and pro-inflammatory response of human neutrophils as well as bacterial phagocytosis by neutrophils through the human formyl peptide receptor 2 (FPR2) [21,32]. Among PSMs, PSM α3 has the most potent pro-inflammatory activity in neutrophils [17,21,32]. Recently, the immunomodulatory properties of α-type PSMs were studied in keratinocytes and on murine models.At a lytic concentration, PSMs are critical in stimulating the release of the pro-inflammatory cytokines IL-18 and IL-1β from human keratinocytes, PSM α3 being the most efficient inducer [26]. In a mouse model of *S. aureus* epicutaneous infection, α-type PSMs were critical in inducing an IL-17-mediated skin inflammatory response associated with epidermal thickening and neutrophil infiltration [33]. In addition, α-type PSMs were shown to trigger the release of IL-1α and IL-36α and subsequent skin inflammation mediated by the MyD88 signaling pathway in both mouse skin and primary human keratinocytes [33,34]. Lastly, α-type PSMs have been reported to induce endogenous serine protease activity such as trypsin activity in primary human keratinocytes, thereby contributing to skin damage in Netherton syndrome [35]. Given that proteolytic activity could contribute to desquamation, reduction of antimicrobial peptide (AMP) expression, reduction of filaggrin expression and inflammation, PSMs may modulate immune response in AD lesions through various mechanisms including induction of pro-inflammatory cytokine and chemokine production from keratinocytes, and play a role in AD pathophysiology [4,9,12,36–38].

This study aims to better understand the impact of PSMs on the human epidermis during *S. aureus*-related skin lesions. For this purpose, we investigated the role of α-type PSMs, especially PSM α3, in the induction of expression and production of a large panel of chemokines and cytokines, known to be involved in the inflammatory response of human skin. Firstly, PSMs produced by the *S. aureus* SF8300 strain were characterized by LC-MS. Then, in order to compare the biological activity of the N-formylated and non-formylated PSM α3, primary human keratinocytes were stimulated with both forms of PSM α3 from *S. aureus* culture supernatants at sublytic concentrations. To explore the role of α-type PSMs among the other virulence factors found in the *S. aureus* secretome, we used supernatants from a wild-type strain producing all PSMs and from mutants deficient in production of α-type PSMs (1 to 4) or all PSMs. Finally, the impact of α-type PSMs in induction of the pro-inflammatory response of human skin was evaluated using *ex vivo* human skin explants, a more physiologically relevant model of pluristratified epidermis.

## Material and methods

### Bacterial strains and growth conditions

The bacteria used in this study, derived from the USA300 strain, were provided by the French National Reference Center for Staphylococci (Lyon, France) and are summarized in Table 1. SF8300 wild-type strain (WT) was used to produce purified PSMs and was grown in Brain Heart Infusion (BHI, Oxoid, Basingstoke Hampshire, Britain) for 20 h at 37°C with orbital shaking at 200 rpm. The SF8300Δpvl expressing all PSMs (Total PSMs), SF8300Δ*pvlΔpsmα1-4* deleted for genes encoding all α-type PSMs (Δ PSMα 1–4) and SF8300Δ*pvlΔpsmα1-4Δpsmβ1-2*Δ*hld* deleted for genes encoding all PSMs (Δ Total PSMs) strains were used for culture supernatant production and were grown in Epilife medium (Gibco, Gaithersburg, MD, USA) in the same conditions as SF8300 wild-type. All strains are deleted for Panton-Valentine leukocidin (PVL) to avoid interference with potent deleterious effects of this toxin expressed by a limited number of *S. aureus* isolates. Cells were centrifugated at 9 000 g for 15 min and supernatants were filtered through 0.22 µm pore size membranes (Sarstedt, KG, Germany) before being stored at −80°C until use.

**Table 1.** **Bacterial strains derived from the USA300 clone and used in this study**. All bacterial strains were provided by the French national reference center for staphylococci [27]

*S.aureus* strain	Genetic background	Description
SF8300 (WT)	USA300-0114	Wild type
Total PSMs	SF8300	Δ*pvlL*
Δ PSM α1-4	SF8300	Δpvl, Δpsm*α1-4*
Δ Total PSMs	SF8300	Δpvl, Δ*psmα1-4*, Δpsm*β1-2*, Δ*hld*

### Purification of PSMs from bacterial supernatants

Using SF8300 wild-type strain supernatants, PSMs were first extracted through liquid-liquid extraction. For 3 supernatant volumes, 1 volume of 1-butanol (Thermo Fisher Scientific, Waltham, MA, USA) was added and shaken in a separating funnel. After 2 h of decantation at room temperature, organic phase was recovered and evaporated at 55°C using a rotavapor (Buchi, Rungis, France) (Supplementary Figure 1). The crude extract was suspended in 5 mL of ultrapure water and pre-purified with a Sep-Pak C18 cartridge (Waters, Milford, MA, USA) using an acetonitril (ACN, Thermo Fisher Scientific) gradient. Sixty and eighty percent of ACN fractions were pooled, evaporated using a rotavapor and resuspended in 1 mL of H_2_O/ACN (1:1, v/v). PSMs in these fractions were purified and separated by reverse-phase HPLC conducted on a Dionex® C18 120 Ȧ (150 x 4.6 mm). The mobile phase consisted of H_2_O/0.1% trifluoroacetic acid (TFA) (buffer A) and ACN/0.1% TFA (buffer B). Elution was monitored at 214 and 280 nm at a flow rate of 1 mL/min using a three-step linear gradient: 40% B for 5 min, from 40 to 100% B over 30 min and at 100% B for 5 min. (supplementary Figure 1b). All the fractions collected were concentrated using a SpeedVac system and suspended in H_2_O/50% ACN/0.2% formic acid. PSMs were identified in each fraction collected by electrospray ionization mass spectrometry (ESI-MS) with a Xevo Q-TOF (Waters) mass spectrometer. MS mass spectra were performed in a positive ionization mode with cone voltage ramping from 20 to 40 V. The source was set to 120°C. Acquisition mass range was performed between m/z 300 to 2000. The *m/z* ratios enable identification of PSMs [39]. The identity of PSM α3 was further confirmed by LC-ESI-MS using the synthetic N-formylated PSM α3 as a standard. This synthetic peptide was used to quantify the amount of PSMs produced by strain SF8300. A calibration curve was performed by measuring the area under the N-formylated PSM α3 peak after injection of a range of concentrations from 31.2 µM to 250 µM (prepared in H2O/50% ACN/0.2% formic acid). Analytical conditions were as described above. Working solutions of purified PSMs were quantified with the Qubit protein assay (Thermo Fisher Scientific) in accordance with manufacturer’s specifications.

### Synthetic peptide

N-formylated PSM α3 (MEFVAKLFKFFKDLLGKFLGNN) was synthesized by Proteogenix (Schiltigheim, France) and assayed to be > 95% pure by HPLC. This peptide was suspended in a H_2_O/ACN (1: 1, v/v) mixture to obtain a concentration of 1 mM and stored at – 20°C until use.

### Human skin samples

The use of human skin samples for research studies was approved by the Ethics Committee Ouest III (project identification code: DC-2014-2109). Written informed consent was given by all subjects in accordance with the Declaration of Helsinki. Normal abdominal or breast skin was obtained from patients undergoing plastic surgery in order to isolate human primary keratinocytes or to cultivate human skin explants *ex vivo*.

### Ex vivo explant culture

For these experiments, normal abdominal skin was cut with a dermatome to obtain a 4 mm thickness flap. After which, 8 mm diameter biopsies were performed and explants were put on a 500 µm nylon mesh filtering (Sefar, Thal, Switzerland) in 12-well plates. Explants were cultivated in Dulbecco’s modified essential medium (DMEM, Gibco) supplemented with 10% of fetal bovine serum (FBS, Gibco) and 1% of penicillin-streptomycin (Gibco) and incubated at 37°C in 5% CO_2_ humidified atmosphere.

### Isolation of human primary keratinocytes from skin samples

After fat and partial dermis removal, the skin was cut into small and thick pieces using scalpel blades and incubated overnight at 4°C in a 2.5 units/mL dispase II solution (Life Technologies, Carlsbad, CA, USA) to gently digest tissues. Epidermal sheets were removed from the dermis using sterile pliers. Then, keratinocytes were dissociated by 0.25% trypsin-EDTA (Gibco) digestion at 37°C. An equal volume of Keratinocyte-Serum Free Medium (K-SFM, Gibco) supplemented with 10% of FBS was added and the cell suspension was filtered through a 40 µm sterile filter before centrifugation at 300 g for 5 min. Keratinocytes were seeded at a density of about 10^7^ cells per 150-cm^2^ tissue culture flask in K-SFM supplemented with 25 µg/mL bovine pituitary extract (BPE, Gibco) and 0.25 ng/mL recombinant epidermal growth factor (EGF, Gibco). The cultures were incubated at 37°C in a humidified atmosphere with 5% CO_2_ until they reached 80% confluence. After a phosphate buffered saline (PBS, Gibco) rinse, the cells were trypsinized with 0.05% trypsin-EDTA and frozen in K-SFM supplemented with 40% FBS and 20% Dimethylsulfoxyd (Sigma-Aldrich, Saint-Louis, MO, USA) in liquid nitrogen until use.

For stimulation experiments, 2 × 10^5^ cells/well were plated in 24-well plates in 1 mL of K-SFM supplemented with BPE and EGF. For cell viability assay, 4 × 10^4^ cells/well were plated in 96-well plates in 0.1 mL of the same medium. When 80% confluence was reached, cells were starved in unsupplemented K-SFM.

### Cell viability assay

Keratinocytes were treated with various concentrations of synthetic PSM Fα3, purified PSM Fα3, purified PSM α3 and bacterial supernatants for 24 h. Cell viability was evaluated using XTT and lactate dehydrogenase (LDH) assays. For XTT assay, XTT labeling mixture was added and cell viability assay was performed using the cell proliferation kit II (XTT, Roche, Basel, Switzerland) according to the manufacturer’s protocol. For LDH assay, supernatants were collected after 24 h and mixed with 500 µL of PBS – 0.1% Triton X-100 (Sigma-Aldrich). Cells were lysed with 1 mL of PBS – 0.1% Triton X-100 and sonicated for 30 s. The LDH released was measured with the Cobas® (Roche) analyzer and viability was calculated, yielding the ratio between the LDH released in supernatants and the total LDH measured in both supernatants and cell lysates.

### Keratinocytes and ex vivo skin explants stimulation

Keratinocytes and skin explants from 3 to 5 patients were exposed to different concentrations of synthetic PSM Fα3, purified PSMs and bacterial supernatants for 3 and 24 h at 37°C in 5% CO_2_. For explants, 20 µL of bacterial culture supernatants were either added to the culture medium to stimulate the basal layer or deposited on the top of the explants using 6 mm filter paper disks to mimic stimulation on the *stratum corneum*. PBS was used for controls. PSM signaling in keratinocytes was investigated with the FPR2 antagonist WRW4 (10 µM, Tocris Biosciences, Bristol, United Kingdom), neutralizing monoclonal Abs against human TLR2 (TLR2 IgA2, 10 µM, Invivogen, San Diego, CA, USA) or the MyD88 signaling inhibitor Pepinh-MYD and Pepinh control (5 µM, Invivogen). Each molecule was added 2 h before the addition of synthetic PSM Fα3. Cell culture supernatants were collected and stored at – 80°C until use, and the cells were lysed in RA1 lysis buffer from the NucleoSpin® RNA extraction kit (Macherey & Nagel, Düren, Germany) supplemented with 3.5 µL β-mercaptoethanol (Sigma-Aldrich). Explants were lysed in 1 mL of lysing buffer containing 300 mg glass beads (diameter ≤106 μm; Sigma-Aldrich) followed by 4 cycles of 20 s at 8 500 rpm using Cryolys tissue homogenizer (Bertin technologies, Montigny-le-Bretonneux, France). All cell lysates were stored at – 80°C until extraction.

### RNA extraction, reverse transcription and real-time PCR analysis

Total RNA was extracted using the NucleoSpin® RNA extraction kit according to the manufacturer’s instructions. RNA concentrations and sample purity were evaluated using the Nanodrop 2000 spectrophotometer (Thermo Fisher Scientific). One µg of total RNA was then reverse transcribed using SuperScript II kit (Invitrogen, Life Technologies). Quantitative real time PCR was performed in 96-well plates using AceQSYBR Green qPCR Master Mix (Vazyme Biotech, Nanjing, China) and the LightCycler 480 system (Roche Diagnostics, Mannheim, Germany). Reaction mixture was composed of 5 µL of Master Mix, 1 µL of forward and reverse primers at 10 µM (Table 2), 2 µL of ultrapure water and 2 µL of cDNA. PCR conditions were as follows: 5 min at 95°C, 40 amplification cycles for 20 s at 95°C, 15 s at 64°C and 20 s at 72°C. Relative mRNA expressions were normalized with 2 independent control housekeeping genes (Glyceraldehyde-Phospo-Dehydrogenase and RPS28 rRNA gene) and reported according to the ΔΔCT method as RNA fold increase: 2^ΔΔ^CT = 2^ΔCT sample – ΔCT control^

**Table 2.** Sequences of primers used for RT-qPCR

Gene	Forward (5ʹ → 3ʹ)	Reverse (5ʹ → 3ʹ)
**CCL-20**	TCCTGGCTGCTTTGATGTCA	TCAAAGTTGCTTGCTGCTTCTG
**CXCL-1**	CCCCTTTGTTCTAAGCCAGA	AAGGCAGGGGAATGTATGTG
**CXCL-2**	GCAGGGAATTCACCTCAAGA	GCCTCTGCAGCTGTGTCTCT
**CXCL-3**	CCACACTCAAGAATGGGAAGA	TCTCTCCTGTCAGTTGGTGCT
**CXCL-5**	GTTCAGGAACCCGCGACCGCTCGCA	CTGTGGGCCTATGGCGAACACTTGCAGA
**CXCL-8**	TTGCCAAGGAGTGCTAAAGAA	AACCCTCTGCACCCAGTTTT
**G3pdh**	GGCTCTCCAGAACATCATCCCTGC	GGGTGTCGCTGTTGAAGTCAGAGG
**IL-1α**	ATCAGTACCTCACGGCTGCT	AACAAGTTTGGATGGGCAAC
**IL-1β**	CTGTCCTGCGTGTTGAAAGA	CTGGGCAGACTCAAATTCCA
**IL-36γ**	TTTGGGAATCCAGAATCCAG	CTCTCTTGGAGGAGGCAATG
**IL-6**	TACCCCCAGGAGAAGATTCC	TTTTCTGCCAGTGCCTCTTT
**RPS28**	CCGTGTGCAGCCTATCAAG	CAAGCTCAGCGCAACCTC
**TNF-α**	TCACCCACACCATCAGCCGCATCG	GGGAAGGTTGGATGTTCGTCCTCC

### Cytokine and chemokine secretion assays

Levels of CXCL8 and CCL20 in cell culture supernatants from explants were determined for each sample using the human IL-8 standard ABTS ELISA development kit (PeproTech, Rocky Hill, NJ, USA) and the human CCL20 ELISA kit (R&D system, Minneapolis, MN, USA) in accordance with the manufacturer’s specifications. Levels of CXCL8, CCL20, TNFα and IL-6 in cell culture from keratinocyte monolayer were determined for each sample using the Milliplex® Human high sensitivity T cell magnetic bead panel (Merck Millipore, Darmstadt, Germany) on the Luminex® analyzer 200 (Merck Milipore).

### Immunohistochemistry

For histology, skin explants were stored in Formol 4% and then embedded in paraffin. Hematein-Eosin-Safran (HES) staining was performed on skin sections by the Department of Pathology of the CHU of Poitiers.

### Statistical analysis

Results were analyzed using GraphPad Prism version 8 (GraphPad Software, La Jolla, CA, USA). The statistical significance of the difference between two groups was evaluated by the one-way nonparametric ANOVA test followed by the Dunn’s test and by the nonparametric Mann–Whitney *t* tests. Differences were considered to be significant when *p* value was less than 0.05.

## Results

### PSM cytotoxicity on primary human keratinocytes

In this study, the strain SF8300, belonging to the USA300 lineage, was used and the characterization of PSMs secreted in growth medium was performed by LC-MS. N-formylated PSM α3 (PSM Fα3) and non-formylated PSM α3 (PSM α3) were strongly produced and purified from bacterial culture supernatant for use in subsequent experiments (Supplementary Figure 1b and 2a-B). PSM γ was also produced, but to a lesser extent (Supplementary Figure 1b and 2c). Relative quantification allowed us to show that PSM α3 is the most abundant form of PSM produced by the strain SF8300. The amounts of PSM α3 and N-formylated PSM α3 produced were approximately 56 µM and 31 µM respectively, as determined by assay in LC-ESI-MS. As this study was focused on the effect of α-type PSMs on epidermis, stimulation of primary human keratinocytes with purified PSM Fα3 and PSM α3 were performed to evaluate a potential biological effect of formylation on skin inflammation as has been previously reported for neutrophil chemotaxis [21]. In addition, a synthetic N-formylated PSM α3 was used as a standard because PSM α3 was previously shown to be the most lytic and pro-inflammatory PSM on various cell types [17,26].

**Figure 1.**
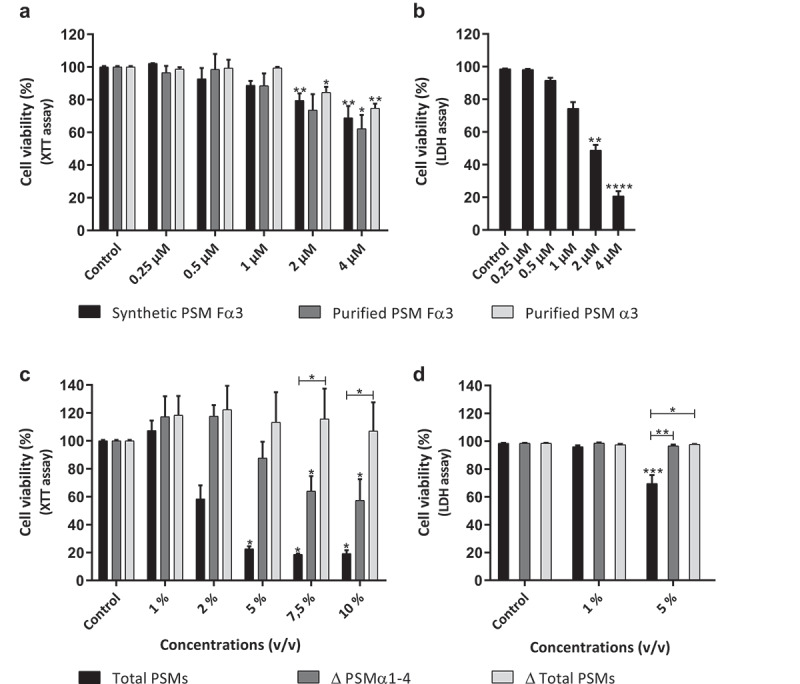
**Cytotoxicity of PSMs on primary human keratinocytes**. Keratinocytes were exposed to synthetic and purified PSM Fα3 (N-formylated) or purified PSM α3 (a-b) and to supernatants from a total PSM-producing strain (Total PSMs), a deleted PSMα1-4 strain (Δ PSMα1-4) or a deleted total PSM strain (Δ Total PSMs) of *S. aureus* (c-d) for 24 h. Cell viability was evaluated using either a XTT assay (a; c) or measuring the lactate dehydrogenase released (b; d). Data are represented as mean + standard error of mean (SEM) of at least three independent experiments. *p < 0.05, **p < 0.01, ***p < 0.001, ****p < 0.0001

First, the ability of these PSMs to induce cytotoxicity on human primary keratinocytes was evaluated using the XTT cell viability assay, which measures cellular metabolic activity. Synthetic and purified PSM Fα3 as well as purified PSM α3 showed a similar effect and caused a significant decrease of keratinocyte viability of about 20% at a final concentration of 2 µM (equivalent to 5 µg/mL) (Figure 1a). To validate our working concentrations, another viability assay was performed for some conditions, the objective being to evaluate induction of cell lysis by PSMs. Thus, LDH release was measured to assess plasma membrane permeabilization by PSMs. The results confirmed that synthetic PSM Fα3 induced significant cytotoxicity starting from a concentration of 2 µM (Figure 1b). After which, to determine the impact of α-type PSMs and other PSMs (among all bacterial secreted factors) on keratinocyte viability, supernatants of a *S. aureus* strain producing all PSMs (Total PSMs) were compared to supernatants of mutants deficient in PSMs α1-4 (Δ PSMs α1-4) and all PSMs (Δ Total PSMs). The absence of expression of specific PSMs in mutant strains was checked by RT-qPCR and no modulation of other toxin expression levels was observed in mutants (data not shown). Supernatants containing all PSMs were highly cytotoxic at a concentration ≥ 2% (volume/volume), leading to decreased viability by at least 40% (Figure 1c). In contrast, supernatants from the mutant deficient for all α-type PSMs were less cytotoxic, inducing a significant decrease of keratinocyte viability from a concentration of 7.5%, whereas supernatant from the mutant deficient for all PSMs was not cytotoxic at the highest tested concentration of 10% (Figure 1c). At a final concentration of 5%, similar results were obtained by LDH release assay, showing that only supernatant containing all PSMs was cytotoxic (Figure 1d). These data suggest that PSMs are major cytotoxic *S. aureus*-secreted factors for human keratinocytes and that α-type PSMs are highly active cytotoxins on primary keratinocytes, acting in a concentration-dependent manner.

### PSMs trigger an intense inflammatory response in primary human keratinocytes

**Figure 2.**
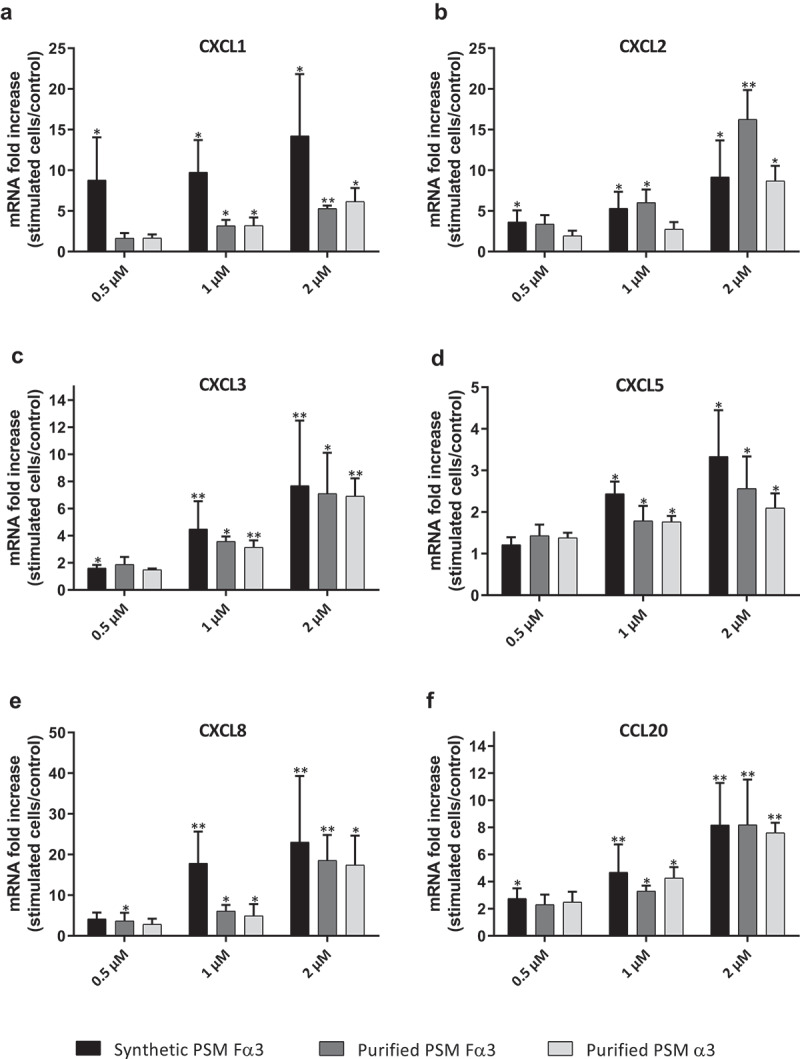
**Synthetic and purified PSM α3 from *S. aureus* triggered pro-inflammatory chemokine expression in keratinocytes at 3 h post-stimulation**. mRNA fold increase of CXCL1 (a), CXCL2 (b), CXCL3 (c), CXCL5 (d), CXCL8 (e) and CCL20 (f) was quantified in keratinocytes at 3 h post-stimulation with synthetic and purified PSM Fα3 or purified PSM α3 as compared to unstimulated keratinocytes. Data are represented as mean + SEM of at least three independent experiments. *p < 0.05, **p < 0.01

**Figure 3.**
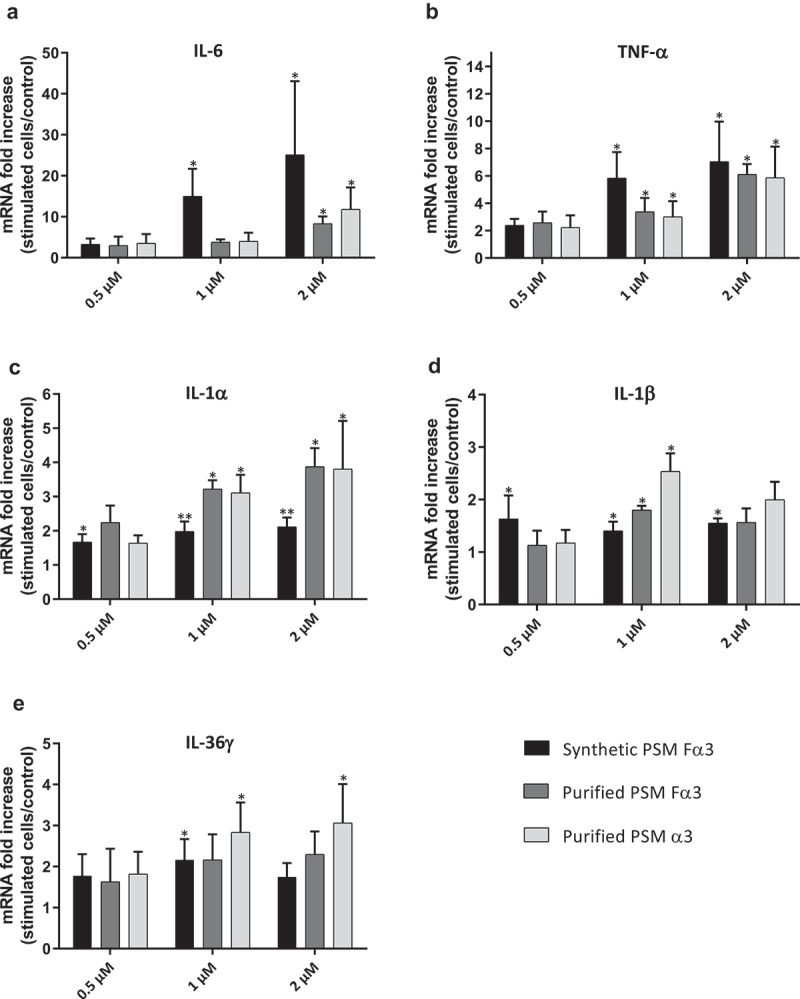
**Synthetic and purified PSM α3 from *S. aureus* stimulated pro-inflammatory cytokine expression in keratinocytes at 3 h post-stimulation**. mRNA fold increase of IL-6 (a), TNF-α (b), IL-1α (c), IL-1β (d) and IL-36γ (e) were quantified in keratinocytes at 3 h post-stimulation with synthetic and purified PSM Fα3 or purified PSM α3 as compared to unstimulated keratinocytes. Data are represented as mean + SEM of at least three independent experiments. *p < 0.05, **p < 0.01

**Figure 4.**
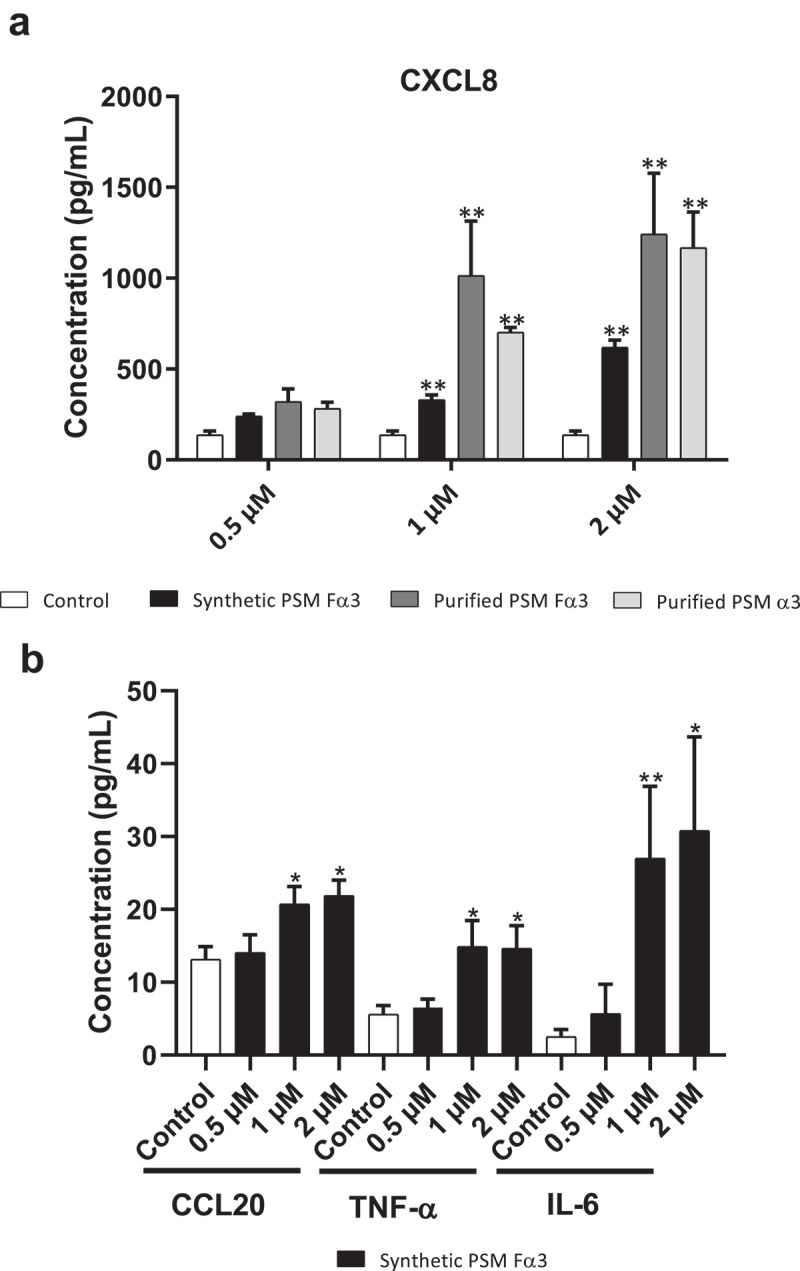
**PSMs from *S. aureus* induced cytokine and chemokine production in keratinocytes at 24 h post-stimulation**. Keratinocytes were exposed to synthetic and purified PSM Fα3 and purified PSM α3 for 24 h. CXCL8 (a), CCL20, TNF-α and IL-6 (b) concentrations was assessed in culture supernatants. Data are represented as mean + SEM of at least three independent experiments. *p < 0.05, **p < 0.01

The ability of PSMs to induce an inflammatory response in primary human keratinocytes was investigated in both aggressive and non-aggressive conditions. Expression of a wide panel of cytokines and chemokines known to be produced by keratinocytes in inflammatory states was assessed following keratinocyte stimulation with non-cytotoxic concentrations of toxins (1 µM and 0.5 µM) and *S. aureus* supernatants (1%) as well as with cytotoxic concentration of α-type PSMs (2 µM). On the one hand, primary human keratinocytes were stimulated with non-cytotoxic and cytotoxic concentrations of synthetic and purified PSM Fα3 and purified PSM α3 for 3 h and 24 h to quantify mRNA expression of pro-inflammatory mediators by RT-qPCR. Similar significantly enhanced expression of the chemokines CXCL1, CXCL2, CXCL3, CXCL5, CXCL8 and CCL20 in cells treated with synthetic or purified PSM Fα3 and purified PSM α3 was observed at 3 h post-stimulation in a dose-dependent manner in both non-cytotoxic and cytotoxic concentrations (Figure 2). This induction of chemokine expression at an early time of stimulation was marked with fold increase varying from 5 to 23 for most chemokines, except for CXCL5, for which a maximum 3.5-fold increase was observed at 2 µM. Similarly, synthetic or purified PSM Fα3 and purified PSM α3 induced significant overexpression of potent pro-inflammatory cytokines by keratinocytes, such as IL-1α, IL-1β, IL-6, IL-36γ and TNF-α, in a dose-dependent manner for concentrations ≥ 1 µM (Figure 3). IL-6 and TNF-α mRNA expression were the most enhanced, with a fold-change ranging from 6 to 25 following stimulation with synthetic PSM Fα3. In contrast, α-type PSMs did not significantly modulate inflammatory mediator expression at 24 h post-stimulation, except for CXCL8, CCL20 and IL-6, for which slightly enhanced expression persisted (data not shown and supplementary Figure 3). These results highlighted fast and transient induction of pro-inflammatory mediators by α-type PSMs. At the proteomic level, both synthetic and purified PSMs Fα3 and purified PSM α3 significantly enhanced CXCL8 secretion levels in a dose-dependent manner as observed at the transcriptomic level (Figure 4a). As purified PSM Fα3 induced higher secreted level of CXCL8 than synthetic PSM Fα3, we cannot exclude the possibility that purified PSMs may be contaminated by trace-level of co-eluting compounds enhancing the peptide pro-inflammatory activity. In addition, synthetic PSM Fα3 also induced a significant release of CCL20, TNF-α and IL-6 in cell culture supernatants from a concentration of 1 µM (Figure 4b).

**Figure 5.**
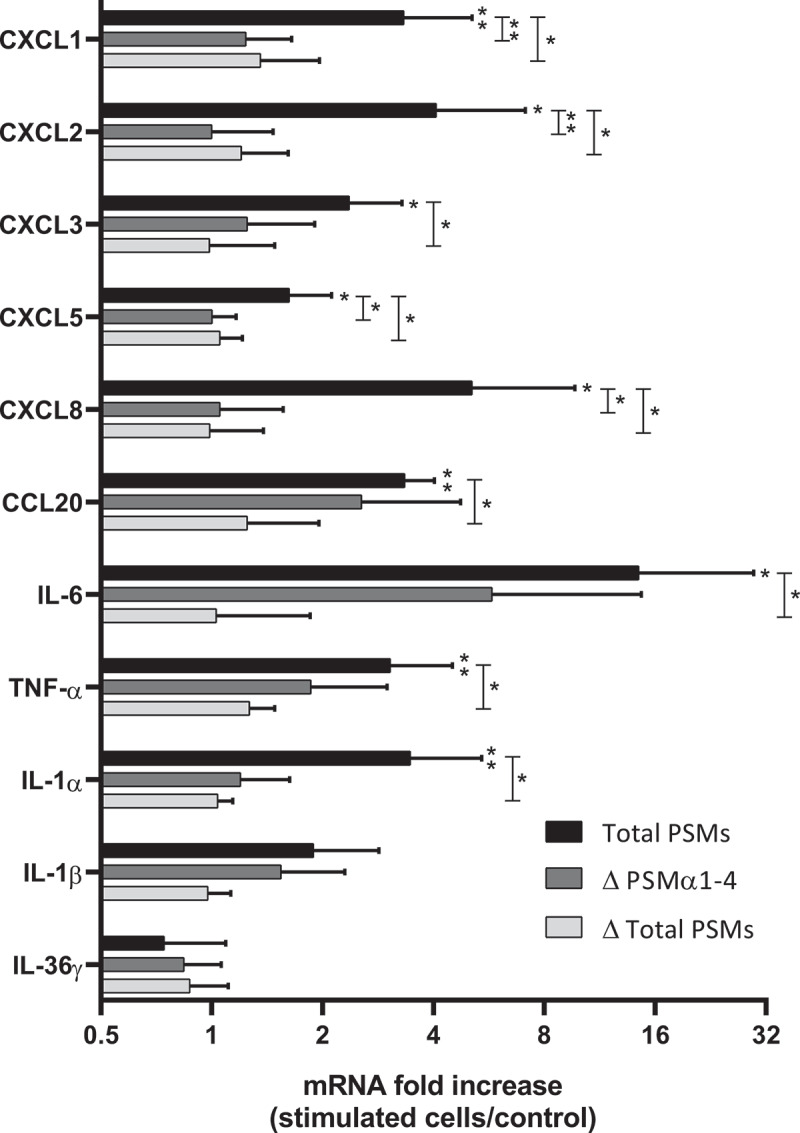
**Secretion of PSMs α by *S. aureus* was crucial to promotion of pro-inflammatory chemokine and cytokine expression in keratinocytes at 3 h post-stimulation**. mRNA fold increase of CXCL1, CXCL2, CXCL3, CXCL5, CXCL8, CCL20, IL-6, TNF-α, IL-1α, IL-1β and IL-36γ was quantified in keratinocytes at 3 h post-stimulation with 1% (v/v) supernatants from total PSM-producing strain (Total PSMs), PSMα1-4-deficient strain (Δ PSMα1-4) or total PSMs-deficient strain (Δ Total PSMs) of *S. aureus* as compared to unstimulated keratinocytes. Data are represented as mean + SEM of at least three independent experiments. *p < 0.05, **p < 0.01

On the other hand, to assess the role of α-type PSMs among other PSMs and secreted *S. aureus* toxins in the induction of inflammatory response, keratinocytes were exposed to the non-cytotoxic concentration of 1% of *S. aureus* supernatants from a PSM productive strain (Total PSMs) and two PSM-deficient strains (Δ PSMs α1-4 and Δ Total PSMs) for 3 h or 24 h. At 3 h post-stimulation, supernatant containing all PSMs caused significant overexpression of a large panel of pro-inflammatory cytokines and chemokines, including IL-1α, IL-1β, IL-6, TNF-α, CXCL1, CXCL2, CXCL3, CXCL5, CXCL8 and CCL20 (Figure 5). In contrast, in keratinocytes treated with supernatants of mutants deficient in α-type PSMs or in all PSMs, chemokine and cytokine mRNA levels were lower or close to basal level of untreated keratinocytes. This finding suggested that PSMs are major pro-inflammatory secreted factors of *S. aureus*. At 24 h post-stimulation, supernatants from the 3 tested-strains did not significantly modify the expression of pro-inflammatory mediators, except for IL-6, for which a 7-fold-increase was observed following total PSM supernatant stimulation (data not shown). Taken together, these results suggest a major role for PSMs, especially α-type PSMs, in induction of the innate immune response of keratinocytes during *S. aureus* infection, leading to overexpression of pro-inflammatory cytokines and chemokines.

The role of the potential pathways previously involved in inflammatory response induced by PSMs was then investigated. In neutrophils, PSMs have been reported to exert their pro-inflammatory action through FPR2 signaling [21]. Nevertheless, no expression of FPR2 mRNA was detected by RT-qPCR in primary human keratinocytes from five different patients used in this study. Moreover, no inhibition was observed in presence of the FPR2 antagonist WRW4, suggesting that this receptor was not involved in the inflammatory response induced by synthetic PSM Fα3 (data not shown). In addition, PSMs have been shown to induce activation of the toll-like receptor (TLR) 2 pathway in human embryonic kidney (HEK) cells [40]. However, use of a human TLR2 blocking antibody did not interfere with pro-inflammatory mediator expression during primary human keratinocyte stimulation with synthetic PSM Fα3 (data not shown). Finally, *S. aureus*-induced skin inflammation also involves the MyD88 pathway, which is required to promote immune responses in keratinocytes and T cells [33,34]. However, addition of the MyD88 inhibitor Pepinh MYD did not abrogate the pro-inflammatory effect of synthetic PSM Fα3 on keratinocytes (data not shown). As a result, FPR2, TLR2 and MyD88 pathways did not seem to be crucial to the PSM α3-induced pro-inflammatory response in primary human keratinocytes.

### PSMs induce pro-inflammatory mediator expression in an ex vivo 3D model of human skin explants

**Figure 6.**
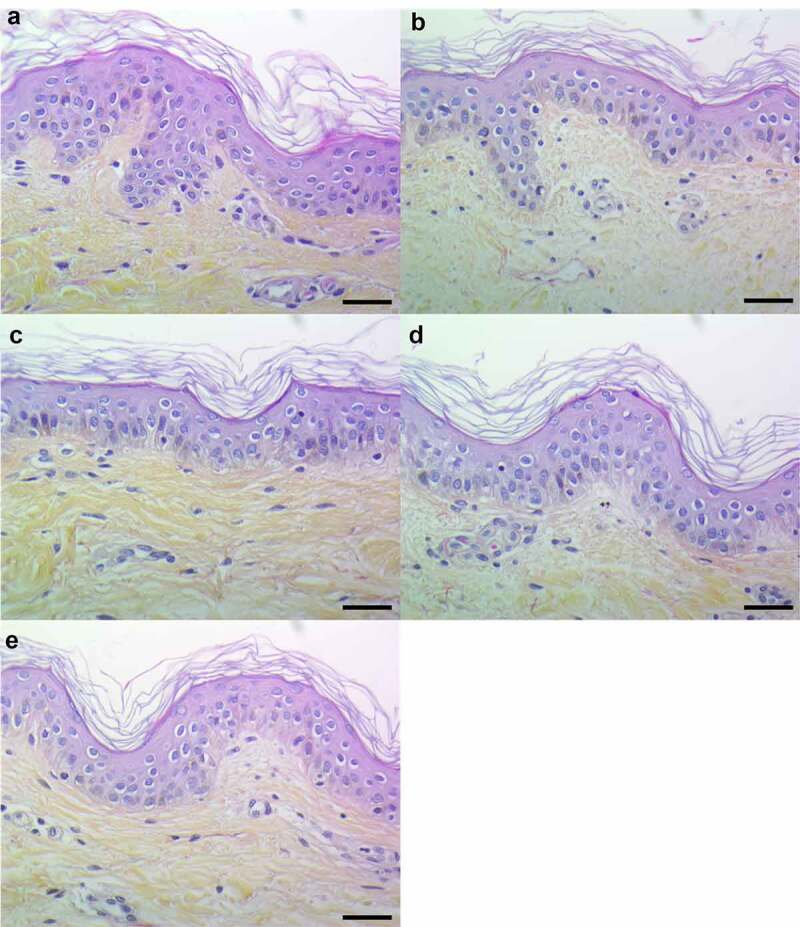
**Histopathology of 3D control and stimulated human skin explants**. Skin sections of explants were stained with Hematein-Eosin-Safran (HES) at 10 min (a) and 24 h post-incubation without (b) or with supernatants containing all PSMs (c), supernatants from PSMα1-4-deficient strain (d) and supernatants from total PSM-deficient strain (e) deposited on a paper disk on the top of explants for 24 h. Scale bar = 20 µm

**Figure 7.**
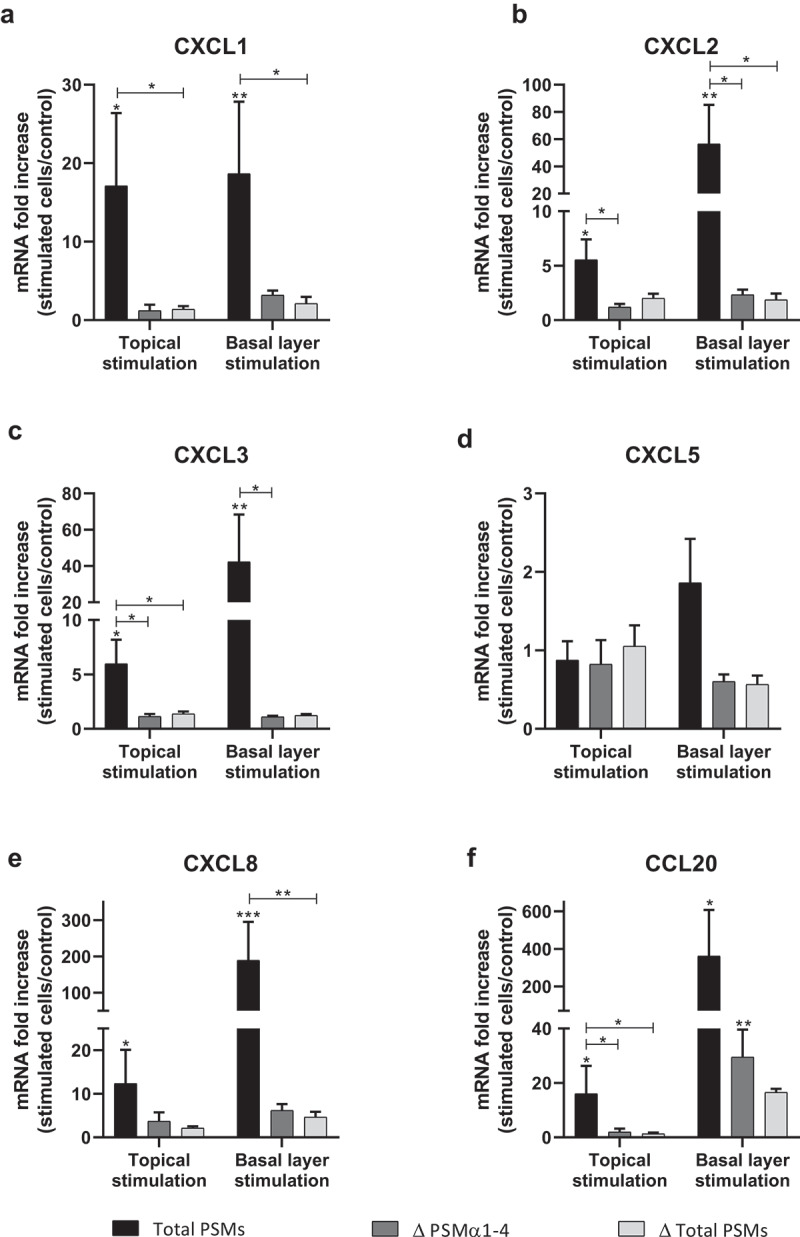
**Topical and basal layer stimulations with PSMs from *S. aureus* of *ex vivo* human skin explants increased the expression of pro-inflammatory chemokines**. Same volume (20 µL) of supernatants from total PSM-producing strain (Total PSMs), PSMα1-4-deficient strain (Δ PSMα1-4) or total PSM-deficient strain (Δ Total PSMs) of *S. aureus* were added directly to the culture medium (basal layer stimulation) or were deposited on a paper disk on the top of explants (topical stimulation) for 24 h. mRNA fold increase of CXCL1 (a), CXCL2 (b), CXCL3 (c), CXCL5 (d), CXCL8 (e) and CCL20 (f) was quantified as compared to untreated explants. Data are represented as mean + SEM of at least three independent experiments. *p < 0.05, **p < 0.01, ***p < 0.001

**Figure 8.**
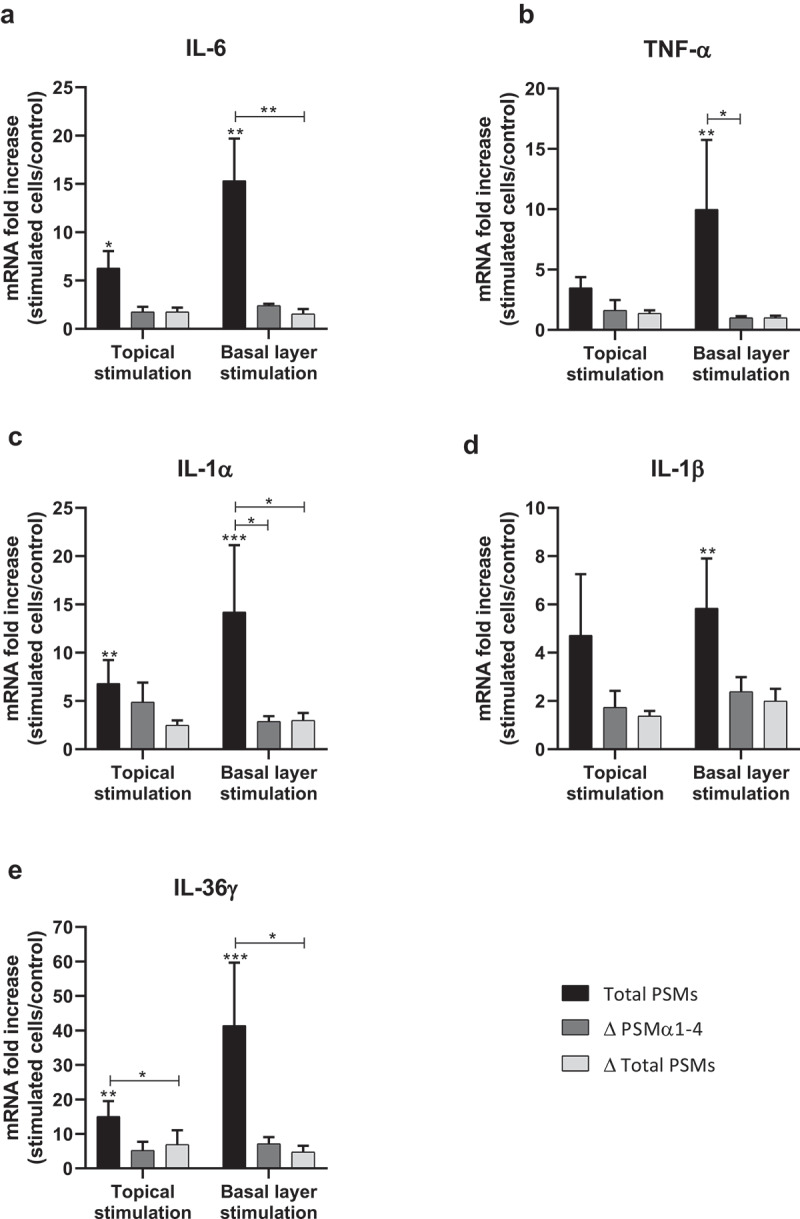
**Topical and basal layer stimulations with PSMs from *S. aureus* of *ex vivo* human skin explants increased the expression of pro-inflammatory cytokines**. Same volume (20 µL) of supernatants from total PSM-producing strain (Total PSMs), PSMα1-4-deficient strain (Δ PSMα1-4) or total PSM-deficient strain (Δ Total PSMs) of *S. aureus* were added directly to the culture medium (basal layer stimulation) or deposited on a paper disk on the top of explants (topical stimulation) for 24 h. mRNA fold increase of IL-6 (a), TNF-α (b), IL-1α (c), IL-1β (d) and IL-36γ (e) was quantified as compared to untreated explants. Data are represented as mean + SEM of at least three independent experiments. *p < 0.05, **p < 0.01, ***p < 0.001

**Figure 9.**
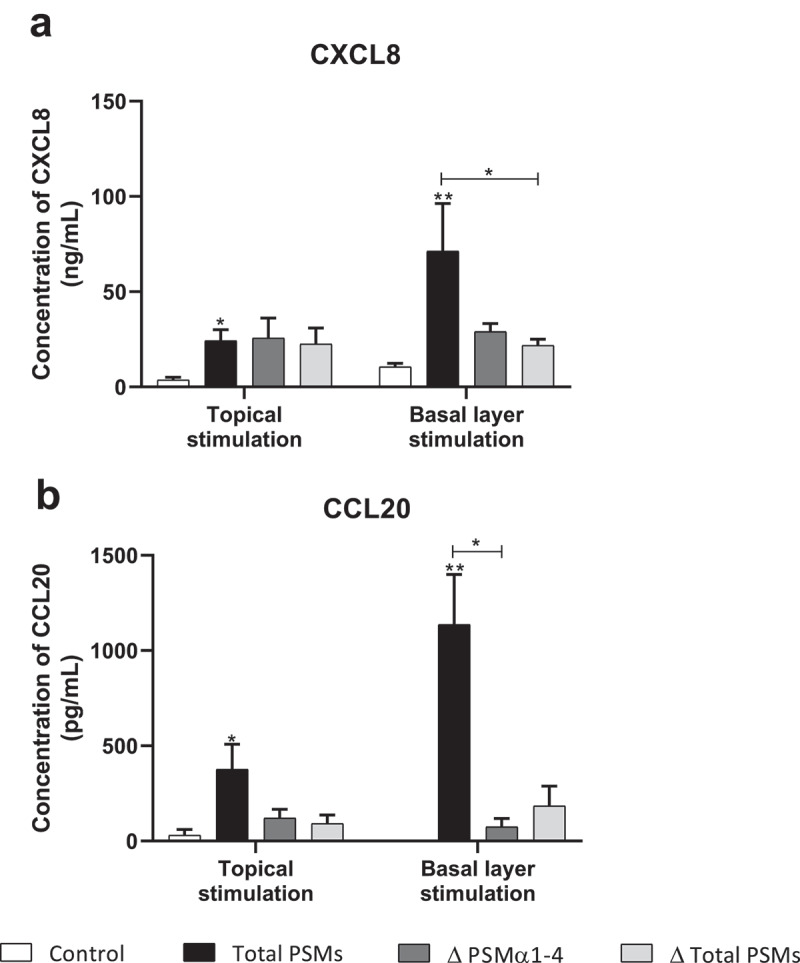
**Topical and basal layer stimulations with PSMs from *S. aureus* of *ex vivo* human skin explants increased chemokine production**. Supernatants from total PSM-producing strain (Total PSMs), PSMα1-4-deficient strain (Δ PSMα1-4) or total PSM-deficient strain (Δ Total PSMs) of *S. aureus* were added directly to the culture medium (basal layer stimulation) or deposited on a paper disk on the top of explants (topical stimulation) for 24 h. CXCL8 (a) and CCL20 (b) concentrations was evaluated by ELISA assays in culture supernatants. Data are represented as mean + SEM of at least three independent experiments. *p < 0.05, **p < 0.01

In order to develop an *ex vivo* model close to an *in vivo* situation and reflecting the architecture of the skin, a 3D model of human skin explants of 4 mm thickness from abdominal plastic surgery was used. In this model, all epidermal layers, from *stratum corneum* to *stratum basal* and part of the dermis were present (Figure 6a) and no alteration of tissue was observed at 24 h post-incubation (Figure 6b). To mimic *S. aureus* cutaneous infection, a topical stimulation of skin explants was performed by depositing paper disk soaked with 20 µL of bacterial supernatant on the *stratum corneum*. In addition, stimulation of basal layer of the skin explants was carried out by adding also 20 µL of bacterial supernatants to the cell culture medium. Both topical and basal layer stimulation with *S. aureus* supernatant containing all PSMs resulted in a marked increase of IL-1α, IL-1β, IL-6, IL-36γ, TNF-α, CXCL1, CXCL2, CXCL3, CXCL8 and CCL20 mRNA synthesis, with fold-increases ranging from 3.5 to 17 for topical stimulation and 6 to 360 for basal layer stimulation (Figure 7 and 8). In contrast, stimulation with supernatants from PSM-deficient strains resulted in slight overexpression of pro-inflammatory mediators, the mRNA levels being markedly reduced as compared with stimulations by supernatant containing all PSMs (Figure 7 and 8). These results were confirmed at the protein level, with ELISA assays showing a similar trend in secretion levels of CXCL8 and CCL20 induced following both topical and basal layer stimulation with the different bacterial supernatants (Figure 9). Interestingly, topical stimulation with 20 µL of synthetic PSM α3 at 100 µM did not induce skin inflammation, suggesting that PSM α3 alone appears unable to cross the skin barrier (data not shown). Nevertheless, we cannot rule out the possibility that PSM α3 may aggregate on the filter paper disk deposited on the *stratum corneum*, leading to loss of pro-inflammatory activity. On the contrary, results obtained through topical stimulation with bacterial culture supernatants highlight the fact that a combination of PSMs with other bacterial secreted factors can diffuse through the *stratum corneum* to stimulate the inflammatory response of keratinocytes. Nevertheless, HES immunostaining from skin section of topical stimulated explants with supernatants containing all PSMs showed the same morphology as control explants and explants stimulated with supernatants from PSM-deficient strains, suggesting that PSMs do not induce alteration of skin integrity (Figure 6c-e). All in all, α-type PSMs appear to be key factors secreted by *S. aureus* involved in induction of cytokine and chemokine produced by a human skin explant model. Taken together, our results suggest an important role for PSMs, especially α-type PSMs, in the pro-inflammatory immune response of human epidermis.

## Discussion

*S. aureus* is a transient resident of human skin, colonizing almost half of the population with persistent carriage in about 20% and intermittent carriage in about 30% [1,2]. *S. aureus* is also responsible for a wide range of infections such as soft tissue infections and invasive infections including endocarditis, osteomyelitis or sepsis [1,2]. Impaired skin and mucosa are the primary sites of *S. aureus* infection, allowing its penetration in deep tissues and the bloodstream. *S. aureus* can not only provoke direct skin infections such as impetigo, cellulitis, ecthyma, abscess formation, but also exacerbate preexisting inflammatory skin diseases such as AD and psoriasis [1,4,41]. Indeed, *S. aureus* is believed to be a key microorganism involved in AD physiopathology, colonizing the lesional skin of almost 90% of patients at a very high density and representing up to 65% of bacteria during AD flares [3,11]. *S. aureus* is known to produce a huge amount of virulence factors, which contribute to its pathogenesis and are suspected of exacerbating inflammatory skin disease [4,9,41,42]. In atopic skin, *S. aureus* toxins may contribute to disease progression e.g., α-toxin may enhance skin barrier impairment, causing the death of keratinocytes, while enterotoxins may cause excessive T cell response. While phenol-soluble modulins may also contribute to pro-inflammatory response but their role in AD pathophysiology remains poorly understood [9,42].

PSMs are abundantly secreted toxins and are considered as major virulence factors for *S. aureus* [16,29]. Since the discovery of PSMs, α-type PSMs (especially PSM α3) have been identified as cytolytic peptides with a strong ability to induce lysis of human neutrophils and erythrocytes, due probably to their amphipathic nature and α-helicity conferring surfacting-like properties [17,25,29,43]. In addition, α-type PSMs can contribute to *S. aureus* resistance to phagocytosis by promoting lysis of human neutrophils following bacterial phagocytosis and by destroying endosomal membranes to avoid lysosomal killing of the pathogen in phagocytes [44,45]. In clinical *S. aureus* strains responsible for persistent bacteremia, high expression of α-type and β-type PSMs was observed and was correlated with neutrophil lysis and enhanced *S. aureus* survival [46]. In human keratinocytes, synthetic PSMs α1-4 and δ-toxin induce significant lysis at a high concentration, PSM α3 being the most potent and leading to the death of almost all cells [26]. This study investigated the role of lower concentrations of PSM α3 in the alteration of human primary keratinocyte viability. In order to avoid potential interferences, the mutant strains used to produce supernatants did not express PVL, a toxin known to exert a strong cytotoxic activity and rarely expressed by skin-colonizing strains. Results obtained with either synthetic or purified PSM Fα3, or purified PSM α3 or with supernatant from mutant strains deficient in α-type PSMs confirmed the ability of these toxins to induce keratinocyte cytotoxicity in a dose-dependent manner. Nevertheless, using supernatants from PSM-deficient strains, deletion of only α-type PSMs did not completely abrogate the observed cytotoxic effect as compared to deletion of all PSMs, a finding suggesting synergistic action of α-type PSMs with other PSMs on keratinocyte viability alteration. While previous studies excluded a role of PSMs β in decreased keratinocyte viability, δ-toxin has been reported to be highly cytotoxic and may play a concomitant role with α-type PSMs in keratinocyte damage [26,33]. Among the *S. aureus* secreted factors, our results highlighted the major effect of PSMs on keratinocyte viability, acting as potent cytotoxins.

Following which, the role of PSMs in pro-inflammatory activity on keratinocytes was studied. Both non-cytotoxic and cytotoxic concentrations of synthetic and purified PSM Fα3, purified PSM α3 and non-cytotoxic concentrations of bacterial supernatants containing all PSMs were shown to significantly induce overexpression of a wide-ranging panel of chemokines such as CXCL1, CXCL2, CXCL3, CXCL5, CXCL8 and CCL20 and cytokines such as IL-6, TNF-α, IL-1α, IL-1β and IL-36γ by stimulated primary human keratinocytes. In contrast, bacterial culture supernatants from mutants deficient in α-type PSMs or all PSMs only weakly modulated the expression of inflammatory mediators. Previously, production of pro-inflammatory cytokines such as IL-18, IL-1α and IL-1β was reported using concentrations of synthetic PSMs and bacterial culture supernatants, which were strongly lytic, inducing more than 80% of cytotoxicity [26,34]. Here, non-cytotoxic concentrations of synthetic and purified PSM Fα3 and purified PSM α3 were also able to trigger the expression of pro-inflammatory chemokines and cytokines, suggesting that a mechanism other than lytic effect was involved in induction of pro-inflammatory mediators. Moreover, an early response of keratinocytes to α-type PSMs was highlighted insofar as upregulation of gene expression occurred after PSM exposure of 3 h and was abolished at 24 h post-stimulation. Nevertheless, contrary to observations on neutrophils [21], no difference was observed between N-formylated and non-formylated PSM α3, excluding an effect of formylation on the pro-inflammatory response in keratinocytes. In addition, convergent results regarding the expression of these pro-inflammatory mediators were obtained using an *ex vivo* human skin explant model, following both topical or basal layer stimulation with bacterial supernatants containing all PSMs, and from mutants deficient in α-type PSMs or all PSMs. In addition, topical stimulation of this *ex vivo* 3D explant model resulted in induction of inflammation, a finding suggesting that secreted factors for *S. aureus*, including PSMs, can diffuse through the *stratum corneum* to target keratinocytes and trigger an innate immune response without altering skin integrity. It was previously shown that extracellular proteases of *S. aureus* are involved in bacterial penetration through the epidermal barrier [47]. As purified PSM α3 alone did not seem to be able to cross the *stratum corneum* to induce skin inflammation, we hypothesize that extracellular proteases contained in bacterial culture supernatants could promote the diffusion of PSM through skin layers. This is in agreement with a recent study suggesting that *S. aureus* protease activity is necessary for PSM α to activate keratinocytes when an intact *stratum corneum* is present [37]. Previously, *ex vivo* human skin explants have been used to study *S. aureus* adhesion and biofilm development [48,49] and our results suggest that it is also a relevant model to mimic epicutaneous stimulation with *S. aureus* virulence factors. Otherwise, our results obtained on *ex vivo* human skin explants were consistent with those obtained on skin of mice challenged with topical exposure of either bacterial supernatants from a α-type PSMs-deficient strain or live *S. aureus* strain deleted for α-type PSMs, showing that α-type PSMs are critical to cutaneous inflammation induction [33,34]. Taken together, our results underline strong pro-inflammatory action of sub-lytic concentrations of α-type PSMs in human skin and suggest a major role for α-type PSMs, most likely due to PSM α3, within *S. aureus* secretome in the cutaneous inflammatory response.

In this study, the proinflammatory action of α-type PSMs observed at sub-lytic conditions could involve other mechanisms than the previously described alarmin release associated with keratinocyte lysis [26,33]. In neutrophils, PSMs exert pro-inflammatory action through the FPR2 receptor. It has been shown that blocking FPR2 with its antagonist WRW4 resulted in a complete inhibition of neutrophil response to PSMs, whereas blocking FPR1 and FPR3 had no effect [21,32,50]. In addition, WRW4 was able to inhibit the mast cell degranulation induced by δ-toxin in a mouse model [51]. In the primary human keratinocytes used in this study, FPR2 was not expressed and the WRW4 inhibitor did not modulate the cytokine and chemokine expression induced by α-type PSMs, thereby suggesting FPR2-independent mechanisms. On the other hand, the involvement of TLR2 and MYD88, a canonical adaptor implicated in this TLR pathway [52], in the keratinocyte response to PSMs was investigated. Previously, a PSM α-deficient strain of *S. aureus* was shown to considerably decrease TLR2 activation and CXCL8 production in TLR2-transfected HEK cells as compared to the wild-type strain [40]. Nevertheless, inflammation driven through the activation of TLR2 was observed only when bacterial extracellular factors, especially lipoproteins were present. Indeed, stimulation of TLR2-transfected HEK cells with synthetic PSM α3 alone did not result in TLR2 activation [40]. To assess the role of TLR2 in response to PSMs in primary human keratinocytes, which are known to express functional TLR2 [53,54], a blocking antibody against TLR2 was added prior to α-type PSMs-stimulation, and no modulation of the inflammation was observed, confirming the previous hypothesis. Lastly, the same observations were made using an inhibitor of the MyD88 protein that was previously identified as a driver of cutaneous inflammation following epicutaneous *S. aureus* stimulation [33,34]. Consequently, our results seem to exclude action of α-type PSMs through FPR2, TLR2 and MyD88 pathways in keratinocytes. To conclude, all pathways and receptors previously identified in other cell types as potent actors in response to PSMs did not seem to be involved in the inflammation triggered in primary human keratinocytes. Further investigations need to be performed to identify the signaling pathways activated by α-type PSMs in primary human keratinocytes.

The ability of α-type PSMs to induce overexpression and secretion of a large panel of pro-inflammatory cytokines and chemokines in human skin could be implicated in exacerbation of inflammatory skin diseases such as AD. Indeed, AD patients exhibit dysbiosis of their skin microbiome with *S. aureus* as the dominant species and *S. aureus* colonization of lesional skin is associated with increased disease severity [3,11]. Our results with human skin explants stimulated by bacterial supernatants suggest that α-type PSMs can diffuse through the epidermis when applied topically to the *stratum corneum*. Among the genes overexpressed by α-type PSMs in keratinocytes, CXCL1, CXCL2, CXCL5 and CXCL8 are powerful neutrophil chemoattractant proteins. These chemokines have been reported to be overexpressed in skin biopsies from AD patients [55–57] and could be implicated in the neutrophil infiltration and inflammation observed in AD [58]. In agreement, reduced neutrophil infiltration in the skin of mice challenged with a topical application of live *S. aureus* deficient for α-type PSMs as compared to mice challenged with live WT *S. aureus* has been reported [33,34]. Consequently, α-type PSMs can enhance neutrophil activation and infiltration in AD lesional skin. In addition, we found that α-type PSMs stimulated CCL20 expression and production. This chemokine, which is associated with the Th17 immune response, is upregulated in non-lesional as well as lesional skin and the blood of AD patients [55,59–61] and may also be implicated in the recruitment of dendritic cells and memory and effector T cells into the dermis of AD patients [62]. Concerning the pro-inflammatory cytokines, keratinocyte stimulation with α-type PSMs resulted in enhanced expression of IL-6, TNF-α as well as IL-1 family cytokines (IL-1β and IL-36γ), which are known to be overexpressed in skin from AD patients and regulate chemokine expression in keratinocytes [55,56,63]. Through induction of various inflammatory mediators known to be upregulated in AD patients, α-type PSMs could thereby contribute to the exacerbation of the inflammatory reactions in lesional skin of AD patients and promote disease severity. Finally, due to their pro-inflammatory properties on epidermis, α-type PSMs could also be involved in other *S. aureus*–associated skin infections.

To summarize, a critical role of α-type PSMs in the inflammatory response of primary human keratinocytes induced by *S. aureus* was highlighted. First, their ability to impair keratinocyte viability at low concentrations was demonstrated. After which, in non-cytotoxic conditions, α-type PSMs appear as major bacterial secreted virulence factors able to trigger pro-inflammatory response in the epidermis. By inducing a large panel of potent chemokines and cytokines known to be highly expressed in skin of AD patients, α-type PSMs may be implicated in the exacerbation of inflammation during AD. Finally, an innovative *ex vivo* 3D model of human skin explants was developed in order to mimic epicutaneous exposure of *S. aureus*-secreted products, which can reflect the skin colonization of the upper layer of human skin occurring in AD patients. This model, closer to *in vivo*, could be used to reproduce the impaired skin barrier or cytokine environment characteristic of AD and, finally, to improve understanding of the role of α-type PSMs in the exacerbation of inflammation during AD. Taken together, our results suggest that α-type PSMs are among the main virulence factors of *S. aureus* secretome during infection of human epidermis with potential deleterious effects on inflammatory skin lesions.

## Supplementary Material

Supplemental Material
