# Supplementary material for: Phenol-soluble modulins α are major virulence factors of Staphylococcus aureus secretome promoting inflammatory response in human epidermis
Source: Virulence. 2021 Sep 13;12(1):2474–92. doi: 10.1080/21505594.2021.1975909 (PMC8451463; doi:10.1080/21505594.2021.1975909)
Supplement: Supplemental Material [file KVIR_A_1975909_SM2102.docx]

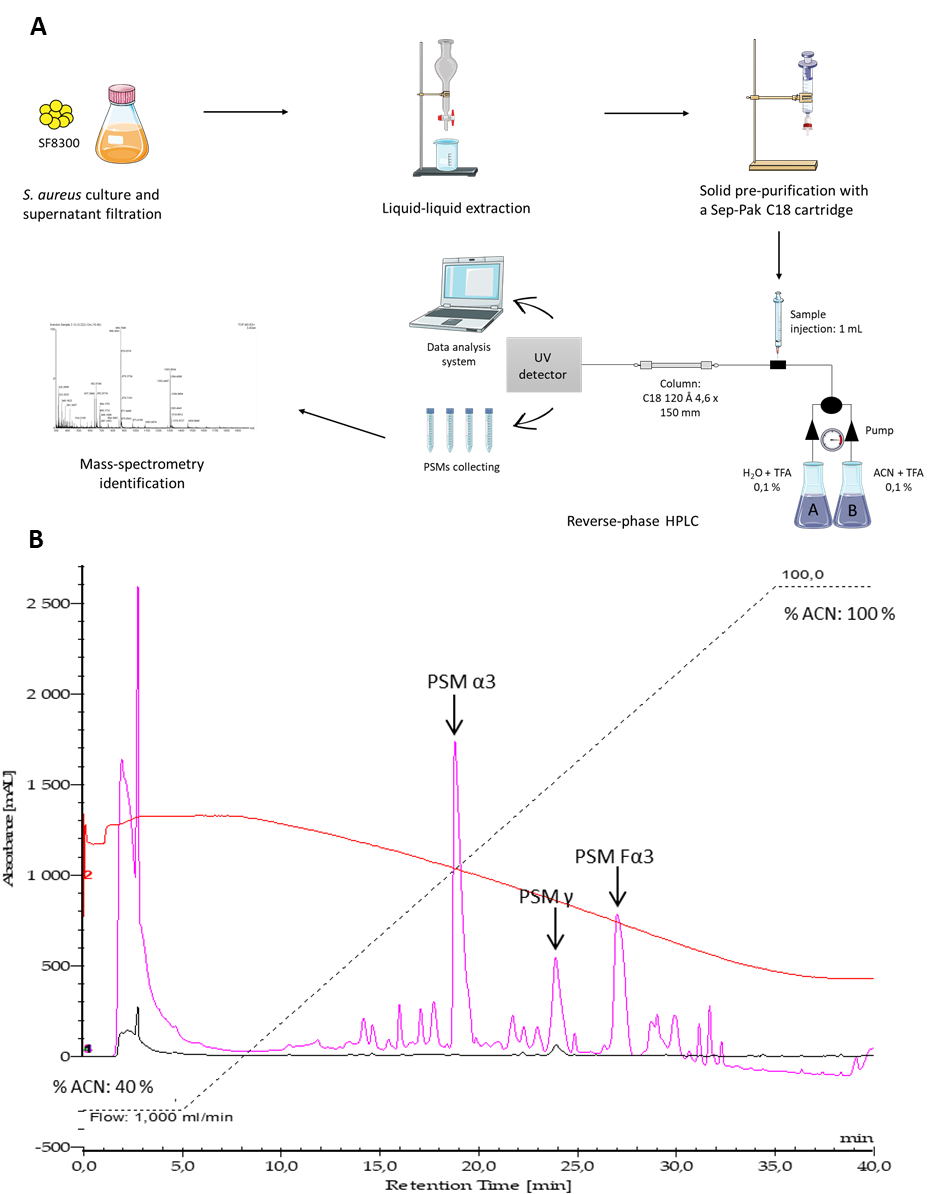


**Supplementary figure 1: Purification and characterization of PSMs produced by *S. aureus* SF8300.** Following *S. aureus* culture, PSMs were extracted with a liquid-liquid extraction, pre-purified with a Sep-pak C18 cartridge and finally purified with RP-HPLC. Collected fractions were identified by ESI-TOF mass spectrometry (A). Reverse-phase HPLC elution profile of PSMs. Detection was carried out by measuring the absorbance at 214 nm (pink line) and 280 nm (black line). Pressure is indicated by a red line. Elution gradient, expressed in % acetonitrile (ACN), is represented by a dashed line (B).


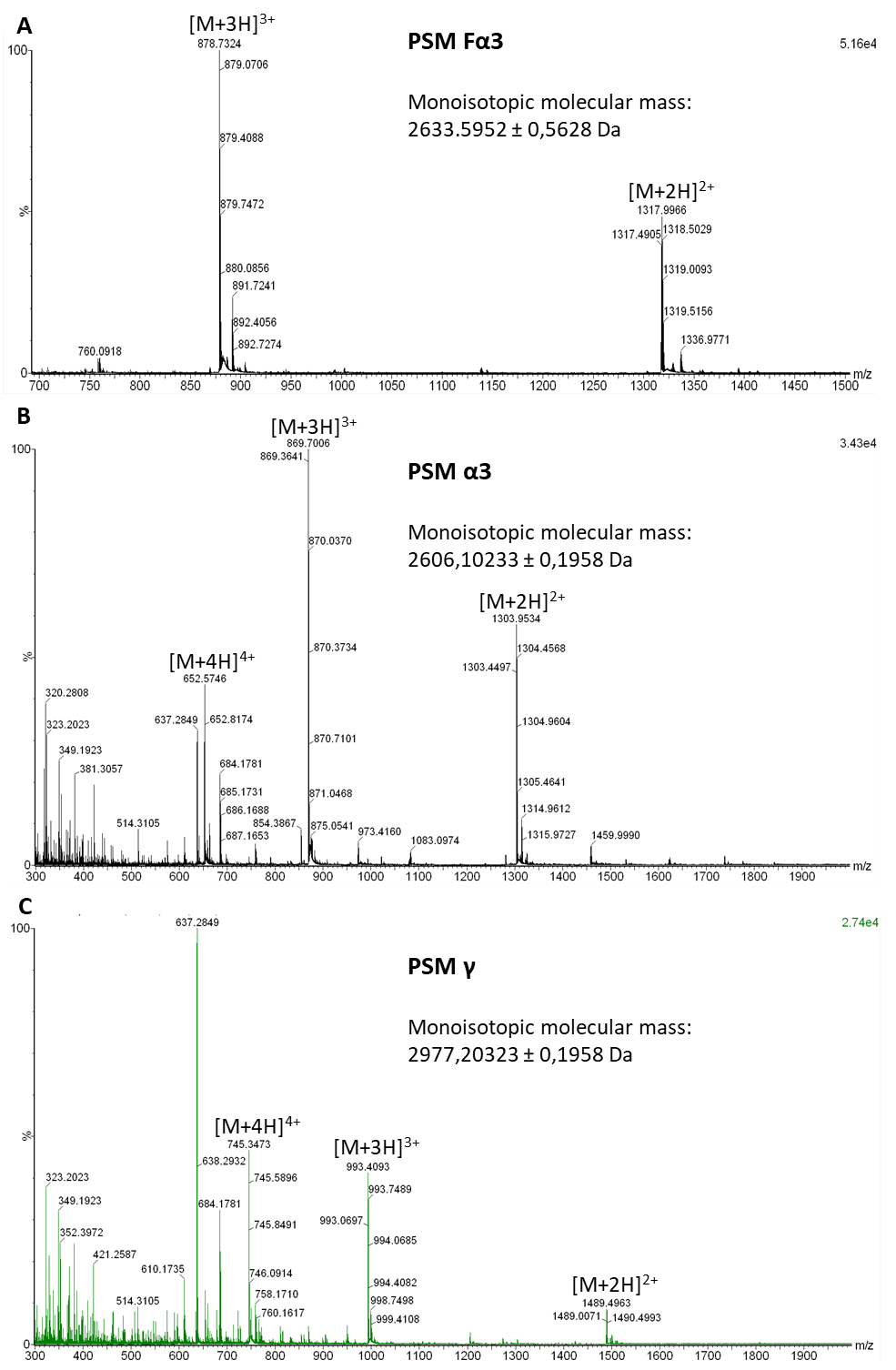


**Supplementary figure 2: PSMs identification after purification.** Pseudo-molecular ions obtained in positive mode from ESI-MS analyses of the purified fraction eluted at 27 min (A), 19 min (B) and 24 min (C) and monoisotopic molecular mass calculated. Main product ions obtained in positive mode from ESI-MS analyses of each purified fraction and identification of PSMs.

**Supplementary figure 3:** **Synthetic and purified PSMα from *S. aureus* slightly increase only CXCL8, CCL20 and IL-6 expression in keratinocytes at 24 h post-stimulation**. mRNA fold increase of CXCL8 (A), CCL20 (B) and IL6 (C) was quantified in keratinocytes at 24 h post-stimulation with synthetic and purified PSM Fα3 or purified PSM α3 as compared to unstimulated keratinocytes. Data are represented as mean + SEM of at least three independent experiments. **p* < 0.05, ***p* < 0.01.
